# Roadkill in a Mediterranean island: Evaluating ten-years of official records

**DOI:** 10.1371/journal.pone.0322644

**Published:** 2025-05-20

**Authors:** Ioannis N. Vogiatzakis, Savvas Zotos, Vassilis Litskas, Stalo Leontiou, Marilena Stamatiou

**Affiliations:** 1 Faculty of Pure and Applied Sciences, Open University of Cyprus, Nicosia, Cyprus; 2 Department of Soil, Plant and Food Sciences, University of Bari Aldo Moro, Bari, Italy; 3 VL Sustainability Metrics LTD, Nicosia, Cyprus.; Tshwane University of Technology, SOUTH AFRICA

## Abstract

Roadkill is a global issue contributing to biodiversity decline which is increasingly recognized by scientists and decision-makers. In Cyprus, a biodiversity hotspot with one of the highest road densities in Europe, the impact of roads on wildlife has been largely overlooked due to the absence of life-threatening collisions. We analysed data from a 10-year roadkill dataset (2013–2022) collected systematically by the Department of Public Works across 27 main roads, primarily aimed at driver safety. Due to the absence of spatial attributes for roadkill locations, the dataset was analysed to identify taxonomic, seasonal, and temporal roadkill trends in Cyprus for the first time. A total of 1,985 roadkill incidents were recorded, involving seven wildlife taxa: foxes (44%), birds (26%), hedgehogs (11.5%), snakes (7%), hares, rats, and lizards. Most roadkill occurred in the Nicosia district (65%). Statistical analyses using chi-square tests with post-hoc Bonferroni corrections revealed strong associations between road types and taxa. Fox and bird roadkill incidents were most common on highways, while hedgehogs and snakes were frequent on secondary interurban or rural roads. Roads near protected areas exhibited higher roadkill frequencies than highways. A positive relationship between traffic volume and roadkill risk was evident, with higher traffic roads posing greater risks. Seasonal patterns showed increased roadkill during spring and summer, peaking in June. This synthesis provides crucial insights into roadkill patterns, offering guidance for conservation and mitigation actions. However, the current monitoring system, focused on driver safety, is inadequate for comprehensive roadkill reporting. Key limitations, such as the absence of spatial data and weaknesses in the protocol, have been identified, with recommendations for improvement proposed to enhance future monitoring efforts.

## 1 Introduction

With the increase of human population worldwide and the expansion of its activities into animal territories, an increase in human-wildlife conflicts (HWC) is recorded in different environmental settings [1,2], operating at various levels [3] which is expected to amplify under climate change [4]. Human-wildlife conflict is a two-way relationship, and while for people it might result in disease transmission, physical attack, and property damage, for animals it means death, habitat pollution, fragmentation, and loss [5].

Although the motivation for HWC studies is usually biocentric, aiming at protecting wildlife from anthropogenic threats [6], in practice the negative effects of these conflicts have been dealt for both humans and animals alike [7,8]. One such manifestation of this conflict is the direct result (i.e., increase of Wildlife Vehicle Collisions - WVC) of the expansion of linear infrastructure in general and roads in particular which, compared to other easily identified and understood manifestations (e.g., land cover change), is insidious and often not evident to policy makers [9] and the public [10].

Although roads play an integral part of economic development, facilitating the movement of people and goods, at the same time they result in irreversible damages to species and habitats [11,12]. The ecological effects of road networks on biodiversity have become an important field of research in the past twenty years [13–15]. Habitat loss and fragmentation due to road construction [16], as well as wildlife loss following collision with vehicles, i.e., roadkill [17], are the main direct impacts of roads on biodiversity.

These impacts are amplified in island environments that are characterised by species population isolation [18,19], low genetic diversity and high endemism [20]. Scientific research focused on roadkill on island ecosystems is limited, with few examples from small-medium islands similar in size to Cyprus [21–24].

Roadkill monitoring, besides recording the direct impacts of roads on wildlife, provides the ability to monitor population trends, map native and invasive species distributions, investigate animal behaviour, and monitor contaminants and diseases [25]. In addition, the value of roadkill monitoring, extends beyond species conservation to traffic safety and economy. In fact, what drives mitigation efforts of fauna casualties in most countries is traffic safety and the economic costs following a collision, which can be substantial [26]. Therefore, several initiatives, usually in the form of Roadkill Observation Systems (ROS), have been set up worldwide in the past 20 years [27,28] to inform decision making in all WVC aspects. These may rely on volunteers/citizen scientists to collect observations [29] or national agencies with an interest in either transport or ecology [30]. In practice, often these initiatives and their resulting systems converge sooner or later into a common hybrid form [31].

In Cyprus, the third largest island in the Mediterranean, the road network has undergone a dramatic increase in the past decades and is currently one of the densest road networks among European countries [32]. Cyprus remains the country in the EU with the highest percentage of soil sealing and land take [33]. Following the global trend of road network expansion, the length of paved roads is projected to increase at a magnitude of 14–23% by 2050 [34]. Cyprus road network is to increase more than 45% in the near future through the design of new highways (122km planned until 2050) when the current highways length is 271km (S1 Fig).

Despite the well-documented effects of road sprawl on wildlife [14], in Cyprus there is only a limited number of studies, which have looked into these effects [35,36]. This reflects the situation in Mediterranean islands in general [24,37], despite the fact that they constitute global biodiversity hotpots [38].

Following a 10-year effort by the Public Works Department, of the Ministry of Transport of the Republic of Cyprus, a dataset is for the first time available which provides an opportunity for roadkill research in this densely road ridden Mediterranean island. The aim of this paper is to provide the first account on the extent of the phenomenon in Cyprus using this long-term data. The main objectives are to a) determine the wildlife group most affected by roadkill along the island’s road network, b) examine wildlife roadkill’s temporal and seasonal patterns, and c) evaluate the longest systematic scheme of roadkill records on the island.

## 2 Materials and methods

### Study area

Cyprus is a biodiversity hotspot [38], hosting a variety of species. There are 30 species of terrestrial mammals (19 of which are bats) [39] including two endemic rodents (*Mus cypriacus, Acomys nesiotes).* The largest mammal on the island is the Cyprus mouflon (*Ovis orientalis*), protected under the Habitats Directive. There are 20 reptiles and three amphibian species, amongst them the endemic and protected, under Annex II of the Habitats Directive, Cyprus whip snake (*Hierophis cypriensis*) [40]. The island is on a migratory route across the Mediterranean and thus important for avifauna at the European and global level [41]. There are 397 bird taxa recorded, 53 permanent residents, including three endemic species (*Sylvia melanothorax, Oenanthe cypriaca, Otus cyprius*) and three endemic subspecies [42].

**Fig 1:**
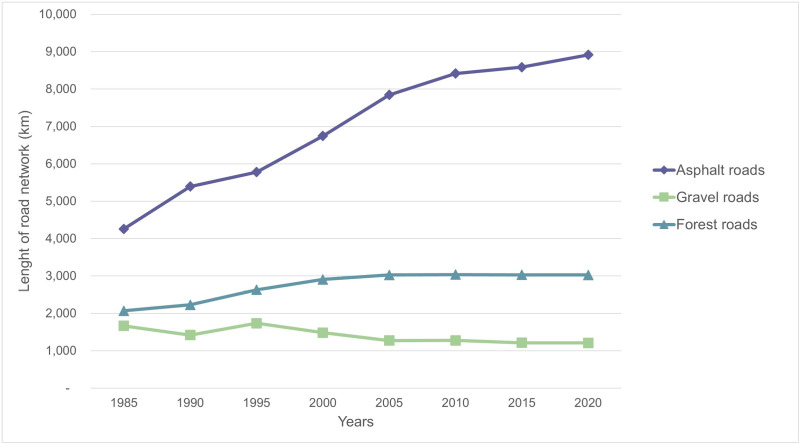
Expansion of road network in Cyprus since 1985. Data from Cyprus Statistical Service [43].

A total length of 19,500 km of roads traverses the island. 68% of the road network (i.e., 13,298 km) is found in the area under the effective control of the Rebublic of Cyprus (5,759 km2). This road network constitutes primarily by asphalt roads 9,074 km (of which 271 km motorways/highways), followed by 3,027 km forest roads and 1,197 km gravel roads [43]. Cyprus road network has dramatically increased in the past decades (Fig 1) and is continuously expanding, with the development of new highways (S1 Fig). Comparing to the population of the island (apr. 1.3 million), Cyprus has one of the densest road network in Europe, equivalent to other Mediterranean countries such as France, Spain, and Greece [32].

### Dataset

**Fig 2:**
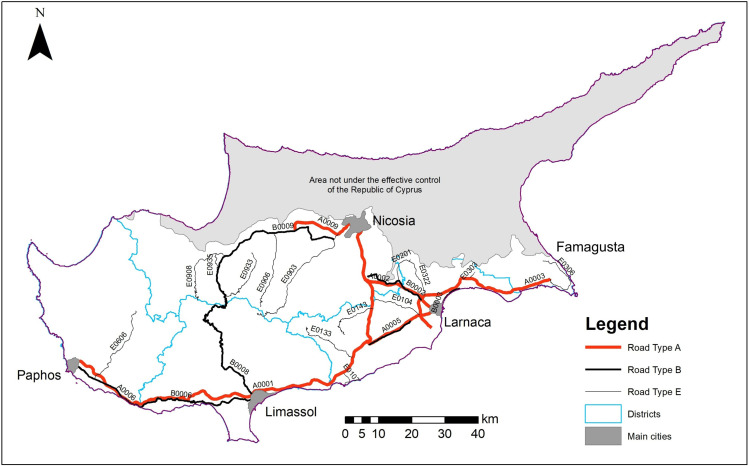
Study area and roads included in the analysis. During the 10-year period (2013-2022) a total of 27 roads (three different types) were monitored by the PWD. Type A: interurban highways (n = 6), Type B: secondary interurban roads (n = 6), Type E: rural asphalt roads (n = 15).

For the purposes of this study, we used a 10-year roadkill dataset (2013–2022) collected by the Public Works Department (PWD). This dataset is part of the systematic effort of PWD to monitor national highways and targeted main roads for the removal of dead wildlife and debris that could cause secondary car accidents. This is the only state-driven effort that address roadkill recording. This effort started in 2013 from the districts of Nicosia and Famagusta, followed by Limassol and Larnaca in 2014. Paphos district was the last to be included in the monitoring scheme in 2017 (S1 and S2 Tables). The initiative, largely focusing on public safety, is a systematic monitoring of interurban highways and selected secondary interurban and rural asphalt roads, that fall under the department’s jurisdiction (27 roads in total – Fig 2). Using the road classification provided by the PWD, the monitored roads fall under Type A (interurban highways), Type B (secondary interurban) and Type E (rural asphalt). These categories are based on road surface type and road width. Road surveys are performed by dedicated and trained personnel of PWD’s district offices using vehicles bearing amber warning lights and driving at low speed (max 60 km/h).

The animals’ carcases are collected and removed from the road following appropriate safety procedures, while PWD officials record the relevant information using a standard protocol. This protocol includes date, road code, detection area (optional), type, and number of roadkill animals detected. The records from the PWD are provided per district.

### Taxonomic groups

As stated, the systematic monitoring scheme of PWD focuses largely on public safety, therefore no proper taxonomical references (i.e., species level) were recorded for the animals detected. The protocols included only four animal categories (i.e., dogs, cats, foxes, and other animals). The rationale for these categories according to PWD was the fact that other large domestic animals (e.g., donkeys, goats) are very rarely, if ever, encountered as roadkill, and if so, they are easily identified and reported by the PWD officers. In addition, Cyprus lacks large wild fauna species, with the exception of a small mouflon population (*Ovis orientalis*), whose individuals were recorded as roadkill mainly on forest roads and not roads under the PWD jurisdiction. Smaller animals (e.g., snakes, lizards, hedgehogs) are not of main concern due to their small size and the extremely low probability of causing a car accident. Nonetheless, in cases where the PWD officials could identify the animal group, they recorded the relevant information in the “other animals” category (e.g., bird, hedgehog, rat, hare, viper). In all other cases where identification was not possible, the roadkill was reported as Non-Identified Animal (NIA).

### Traffic volume – speed limit

Traffic volume data were extracted from the Annual Census of Traffic of 2021 provided by the PWD, as the average traffic volume per road segment. Vehicle speed was not directly examined in this study. Speed limit per road type was used instead as a surrogate of vehicle speed per road section. The speed limit on the roads examined ranges from 50 km/hr to 100 km/hr (Table 1).

**Table 1:** Number of roadkill recorded by the PWD, during the 10-year period (2013-2022) in the 27 roads monitored. Roadkill incidents are shown by animal group (taxon) and in total. NIA = Non-Identified Animal.

Road code	Speed limit (km/hr)	Taxon						NIA	Total roadkill	Road length	Roadkill/km/year
**Fox**	**Bird**	**Hedgehog**	**Snake**	**Rat**	**Hare**
A1	100	165	131	6	23		3	50	378	168.85	0.22
A2	100	131	66	1	4		1	8	211	52.66	0.40
A3	100	150	25		4			22	201	108.8	0.18
A5	100	37	9		1		2	7	56	48.15	0.12
A6	100	79	6		1		7	8	101	121.01	0.08
A9	100	54	92	10	6	4		10	176	38.36	0.46
B2	65	1							1	21.31	0.00
B3	65	2							2	9.92	0.02
B5	65	4							4	14.84	0.03
B6	65	4							4	70.38	0.01
B8	65	9						1	10	44.39	0.02
B9	65	25	51	147	43	35	1	6	308	67.17	0.46
E104	50	4							4	15.58	0.03
E107	50	1							1	5.63	0.02
E133	50	1					2	1	4	21.31	0.02
E143	50	1							1	19.72	0.01
E201	50	6	2						8	9.52	0.08
E303	50	1							1	2.37	0.04
E306	50	1			2				3	22.76	0.01
E322	50	35	1		2				38	12.37	0.31
E606	50	2							2	29.31	0.01
E611	50	2							2	0.44	0.45
E903	50	1							1	45.14	0.00
E906	50							1	1	26.85	0.00
E908	50	5	1	13	12	1			32	29.66	0.11
E933	50				2				2	23.92	0.01
E935	50		2			1			3	8.50	0.04
**Total**		**721**	**386**	**177**	**100**	**41**	**16**	**114**	**1,555**		

### Data analysis

We analysed all records that were allocated to road names/codes. Records which had multiple road codes allocated to them, were removed from the analysis due to absence of a unique spatial attribute. Where only the name of the district was given to an incident, we assigned it to the district’s main highway, as advised by the PWD. We examined the distribution of roadkill per year, season, road type, and animal group.

We tested for possible differences in roadkill per km of the principal roadkill taxa (foxes, hedgehogs, birds and snakes) in road types (A, B, E), traffic volume (high, medium, low) and season (spring, summer, autumn, winter) using chi-square tests, with post-hoc Bonferroni correction to identify statistical differences between pairs of groups. To assess the relationship between traffic volume and roadkill, we used Spearman’s rank correlation test. The analysis was based on 19 of the 27 roads monitored for which data on traffic volume were available. All analyses were conducted in R version 4.3.2 [44].

## 3 Results

### Roadkill summary

A total of 14,895 incidents were recorded over a 10-year period. Out of these, 1,985 involved wild animals and can be considered as roadkill, while the rest involved cats (n = 9,323) and dogs (n = 3,587). Most of the wildlife incidents were recorded in Nicosia district (n = 1,297; 65.3%) followed by Larnaca district (n = 450; 22.7%) and Limassol (n = 159; 8%), with only 66 (3.3%) from Paphos and 13 (0.7%) from Famagusta district.

### Roadkill per taxonomic group

Out of 1,985 roadkill, the majority were foxes (44%), followed by birds (26%), hedgehogs (11.5%), and snakes (7%). A small percentage (4%) were identified as hares, rats and lizards while c. 8% of the incidents were reported as Non-Identified Animal (NIA). For birds, 325 roadkill incidents were identified, with the most frequent species being doves/pigeons (48%), owls (18%), and hooded crows (16%) (S3 Table).

### Roadkill per year and season

The average number of roadkill per year was 198 (SD = +/- 103). The highest number was recorded in 2014 and 2015 (over 300 incidents), while the years 2018 and 2019 have the lowest recorded number of roadkill (under 100). During the COVID years, 2020 and 2021, number of roadkill were below the 10-year average (S1 Table).

**Fig 3:**
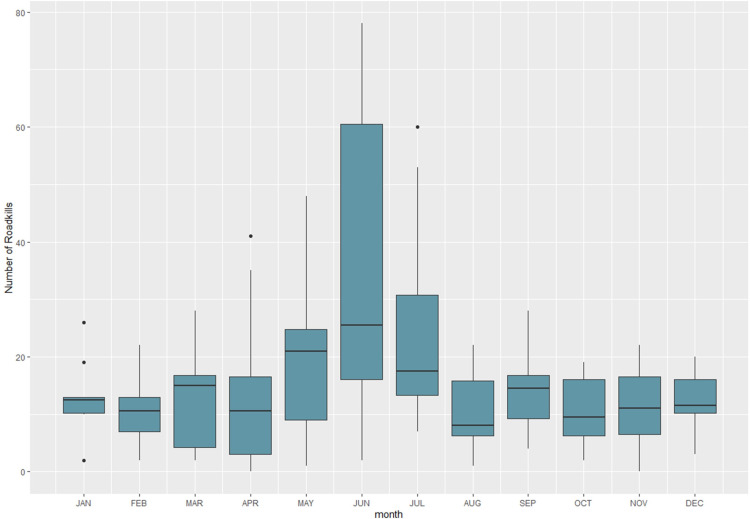
Number of roadkill incidents recorded by the PWD per month during the 10-year period (2013-2022).

**Fig 4:**
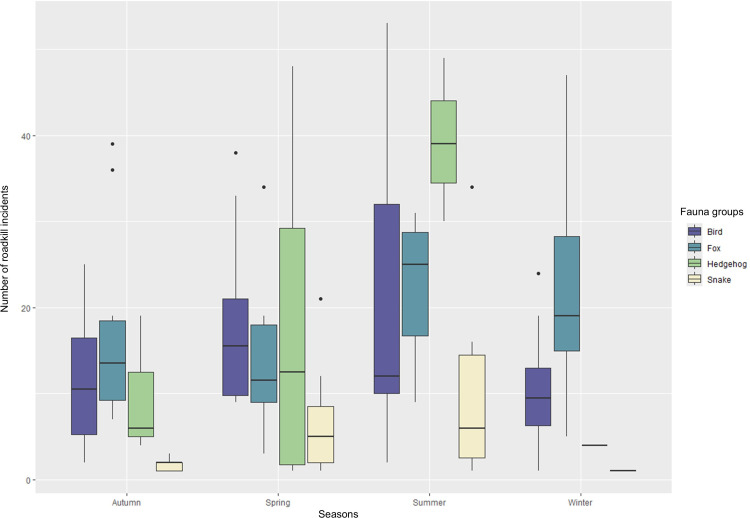
Number of roadkill incidents recorded by the PWD per season during the 10-year period (2013-2022). Roadkill incidents are categorized by fauna group for the four most frequently recorded groups.

There is a clear seasonal increase during spring and summer months peaking in June when most of the incidents are recorded (Fig 3). Chi square test showed that there is a significant association between examined taxa and seasonality (x^2^ = 165.72, p < 0.001). Bonferroni corrections confirmed a strong association between most taxonomic groups and seasons, with the exception of birds (Fig 4).

### Roadkill per road type and traffic volume

From the 1,985 identified roadkill incidents, only 1,555 contained spatial information in the form of the road code and/or description of the locality in a way they could be further statistically analysed (Table 1).

**Fig 5:**
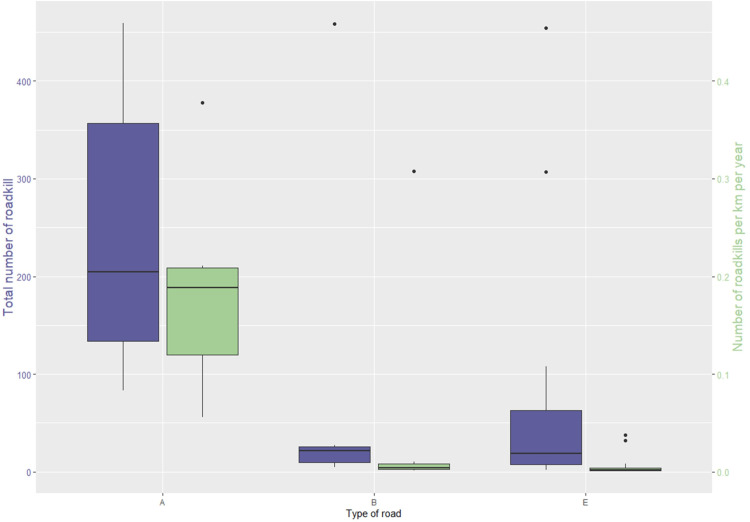
Total number of roadkill and number of roadkill per km per year for the types of roads examined. Type A: interurban highways, Type B: secondary interurban roads, Type E: rural asphalt roads.

**Fig 6:**
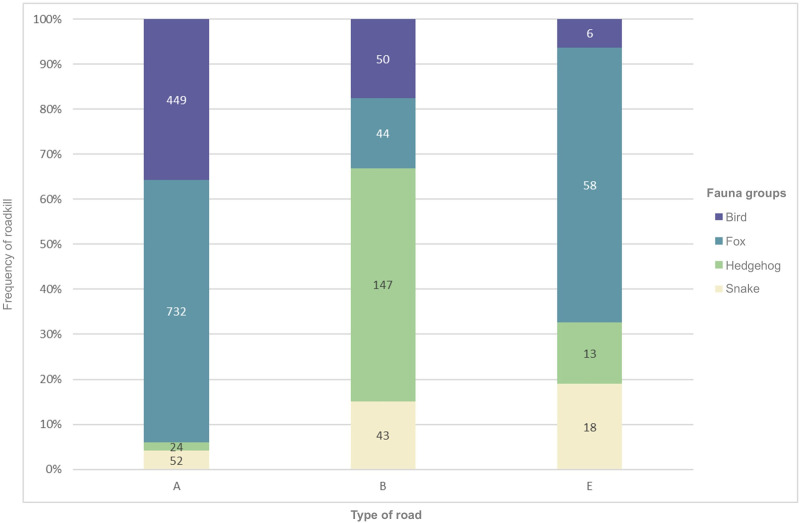
Frequency of roadkill per type of road. Roadkill incidents are categorized by fauna group for the four most frequently recorded groups. Type A: interurban highways, Type B: secondary interurban roads, Type E: rural asphalt roads.

Most of the roadkill incidents occur on the main Highway (A1) followed by Β9, A2 and A3 (S2 Fig). A strong difference can be seen between the road types and the number of recorded animals (Fig 5). In road type A (A1-9), foxes are the most common category followed by birds. Road type B (B9), is by far dominated by hedgehogs, while E roads (E322 and E908) have a much lower frequency of roadkill than the rest of roads (Fig 6), although the percentage of foxes are similar to the ones recorded in road type A.

**Fig 7:**
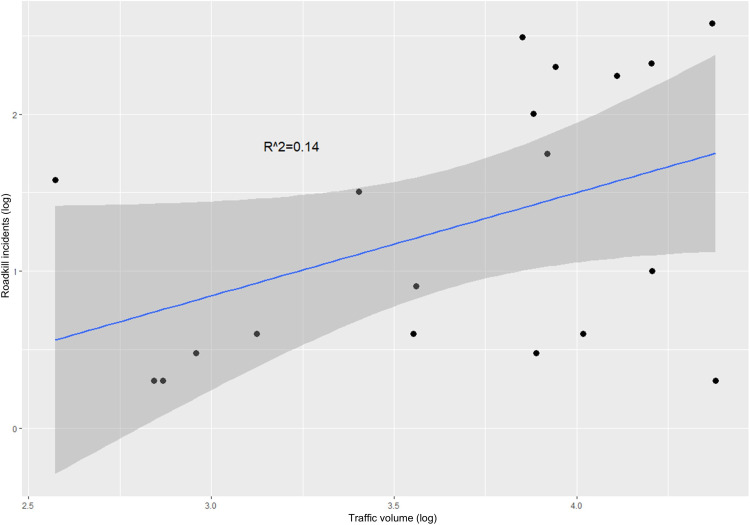
Relation between total roadkill and traffic volume for the roads examined (both axes in logarithmic scale).

A chi square test on the examined roads shows that there is a significant association between the main taxa and road type (x^2^ = 22.407, p = 0.0046) as well as main taxa and traffic volume (x^2^ = 13.103, p = 0.0255). Bonferroni corrections confirmed a strong association between foxes and type A and B roads, while for birds and snakes there is a strong association with Type A roads only. The number of total roadkill showed a positive correlation with traffic volume (Pearson correlation r^2^ = 0.14, p = 0.01) (Fig 7).

## 4 Discussion

This is the very first report of roadkill from Cyprus based on a systematic, ten year long, monitoring approach. Following a decade of records, there have been c. 15,000 roadkill incidents of which 1,985 (13.3%) are wildlife and 12,910 (86.7%) domestic animals (cats and dogs). The impressive dominance of domestic animals over wildlife in the dataset, could be attributed to: the high densities of feral cats on the island (near a million according to animal welfare organizations), the difficult detection of smaller animals on the road surface [45,46], or the quick removal of small carcases by other animals [47]. We acknowledge that domestic animals are more likely to cause a life-threatening accident in Cyprus, since the island lacks large ungulates or carnivores, therefore this official monitoring scheme was developed principally for road safety reasons.

The only large wildlife animal that has been reported as roadkill under this monitoring scheme were foxes, which remain, due to their size, on the road for longer periods [48] and are more likely to be removed by the authorities for safety reasons [49]. Roadkill of the larger wildlife animal on the island, the Cyprus mouflon, has only been reported from forest roads near Pafos forest [50] which are not part of this monitoring scheme. The majority of small-sized roadkill under the current monitoring scheme were birds (mainly doves/pigeons and owls), hedgehogs (only one species exists in Cyprus – *Hemiechinus auritus*) and snakes (unidentified at the species level).

The reduction of roadkill during COVID reported herein, is in agreement with similar studies in Europe and beyond [51–53] as a result of limited road use and, therefore, traffic volume for a significant period of time.

Large animals, including domestic ones were more likely to be reported given their potential impact on traffic and safety. Small animals, such as lizards and hedgehogs, when found in high traffic roads, were more easily neglected by PWD personnel since removing them from roads is more dangerous than leaving them in place. This kind of recording biases need to be considered carefully when drawing conclusions on wildlife conservation from monitoring schemes designed primarily for traffic safety [28,54].

### Behavioural response

The behavioural responses of wildlife to roads and traffic are species-specific and depend on a number of behavioural properties and biological traits [55–57]. Susceptibility to roadkill might be the result of large movement ranges/requirements [58], or opportunistic behaviour in search of food [59].

In our study, the higher roadkill rates in foxes could be attributed to foraging (hunting/scavenging) behavioural traits and territorial requirements [60]. For birds, the most frequent species groups represented among roadkill include doves/pigeons, crows and owls. This is related to the scavenger behaviour of doves and corvids along road verges that is associated with higher roadkill risk in urban settings [47,61]. For the case of owls, high roadkill numbers are associated with their nocturnal activities and related foraging behavioural adaptations [62] such as low flight height, and low foraging speed [63]. The direct impact of roads on owls is well-documented [64].

Similar behavioural responses to roads, linked to foraging activities, have also been observed for hedgehogs. Their nocturnal ranging behaviour within the urban setting and their slow pace make them prone to becoming roadkill [65]. With roads becoming barriers to hedgehog’s movements [66] roadkill is among the most important factors for the decline of hedgehogs in Britain [67].

Herpetofauna has the highest levels of road mortality [68]. Snakes are usually unable to evade traffic, they are not conspicuous to on-coming drivers [69], and intentional kills primarily due to fear (ophiophobia) are not uncommon [70,71].

### Road type and traffic volume

In addition to the behavioural responses, various road-related factors contribute to an increased risk of roadkill such as road type (width, barriers), traffic intensity, road adjacent habitats, and road density [72,73]. Our data show a strong and clear differentiation between the taxa recorded in different road types, which is statistically corroborated. This differentiation is quite sharp between interurban highways (type A) and secondary interurban (type B) or rural asphalt roads (type E). Large size roadkill (e.g., fox and bird) “dominate” the island’s highways, while smaller animals (e.g., hedgehogs and snakes) are the most frequently reported animals on secondary inter-urban roads or tarmac rural roads. This can relate to both the behaviour of PWD’s personnel conducting the monitoring as well as the animals’ response to the type of road. In the first case, the small size of the animals and the low carcass persistence makes it difficult to locate the carcase on the high speed intercity highways [45,46]. Even if located, due to the minor risk for secondary collisions, the recorders might choose to ignore it rather than undertake the dangerous task to record and remove it [30]. In the second case, smaller animals tend to completely avoid highways or remain on road verges instead of crossing the roads and become roadkill [74].

One of the most important road-related factors are vehicle speed and traffic volume [75,76]. Traffic volume, a key factor affecting wildlife on roads, limits animals’ movements or results in mortality, reduced population abundance and species composition [64,77]. The frequency of total roadkill showed a positive correlation with traffic volume, implying that the risk of being roadkilled is greater on higher traffic volume roads (e.g., Highway A1), which is consistent with similar studies elsewhere [e.g., 78,79]. This is an important finding considering the Cyprus government plan for further highway expansion (122 km until 2050 – see S1 Fig). The reason is that on large highways, the direct impact of WVC, is amplified by the indirect, more severe, impact to normal migration patterns and interruption of reproduction [80,81]. The larger the highway the wider the impact from the road effect zone [82]. An impact that is related both to high traffic volume but also to the road technical characteristics it has been revealed during periods of low traffic (i.e., COVID period) [83]. This indirect impact of highway on the wild fauna of Cyprus, has never been studied before. Due to the complete absence of properly designed overpasses and underpasses on the island, highways constitute an impenetrable barrier for small animals (e.g., hedgehogs, snakes) with direct impact on population distribution and dynamics [84–86], currently neglected by the environmental authorities.

### Seasonality

Since traffic volume is fairly constant throughout the year, with the exception perhaps of certain festive periods, the increase in recorded roadkill during spring is linked to the species’ ecology and behaviour [87,88]. Temporal variations in roadkill indicate different biological periods that influence animals’ activity [89,90]. Particularly during spring, with the beginning of the breeding season for most species, there is an increase in their movements and activity. These movements are linked both to their ecological needs (e.g., increased nutritional requirements, thermoregulation), and to their reproductive behaviour (search for mate, set territories, secure oviposition sites, safe havens, and nests). With the end of the breeding season, the onset of winter, and the difficulty of finding food, the activity of individuals is generally restricted. Exothermic animals in particular choose to limit their activity during the winter period to a minimum, avoiding long movements.

### Protected species and protected areas

Most of roadkill incidents are located on the A1 (Nicosia – Limassol) and B9 (Akaki – Astromeriti) highways. It is interesting to note that although the figures representing the total roadkill are similar (~350 individuals in total), the roadkill number per km/year on road B9 is two times that of A1 (0.46 roadkill/km/year versus 0.22 roadkill/km/year). At the same time, two more roads present high ratio of roadkill/km/year. Those are the A9 (Nicosia - Troodos) and A2 (Nicosia - Larnaca) highways (0.46 roadkill/km/year and 0.40 roadkill/km/year respectively). This is an important finding since part of these roads go through or near important Natura 2000 sites (e.g., Paphos Forest, Troodos National Forest Park). Despite the fact that these areas are strictly protected by national and European legislation (i.e., Birds and Habitats Directives), there has been only one roadkill mitigation attempt related to the endemic snake (*Hierophis cypriensis*) which has not yet been evaluated [91]. A variety of mitigation measures, such as calming devices, eco-passages and wildlife fencing, have been successfully implemented worldwide within protected areas and could be also considered in Cyprus [92,93].

Protected areas host species populations of conservation importance that can become roadkill in their effort to colonize nearby areas [94–96]. Therefore, this work aligns with the worldwide concern about the expansion of roads and their impacts within protected areas, which host a great number of important species [92] but also to earlier work on Cyprus which has highlighted that road density within protected areas is as high as outside (2.2 km/km^2^) [32].

There were no roadkill records of highly protected species in the dataset. However, in the last ten years the Game and Fauna Service (GFS) has recorded 15 roadkill incidents involving mouflons, suggesting that road monitoring should also be expanded to more remote areas and different road types. Similarly, there are numerous opportunistic reports, from both competent authorities and citizens, on roadkill species of EU concern (Birds and Habitats Directives) such as the endemic Cyprus whip snake (*Hierophis cypriensis*) [97].

### Monitoring scheme

The dataset by PWD is the only systematic long-term source of roadkill on the island. However, the monitoring scheme needs further improvement:

i) it covers mainly highways and few selected main roads under the jurisdiction of PWD, and although conducted on a daily basis our analysis revealed that this is done in an inconsistent manner;
ii) PWD personnel find it difficult to identify some roadkill groups (e.g., snakes, birds) to the species level;
iii) the absence of roadkill’s positional information makes it difficult to apply any accurate geospatial analysis;
iv) small size animals are underreported, possibly due to their limited influence on road safety.

Government departments working in nature conservation who have the taxonomic knowledge (Department of Environment, Department of Forests, Game and Fauna Service), despite their interest in the phenomenon, are currently not monitoring roadkill systematically as it is done in many countries worldwide [27,28,31]. Of course, it is not uncommon even in these countries for official roadkill data, provided by various sources (police, government departments, hunters, NGOs), to be collected in a non-systematic manner and not designed to study wildlife-vehicle conflict [54]. Cyprus is not an exception.

State initiatives on roadkill, which focus principally on road safety, record Wildlife-Vehicle Collisions (WVC) when the outcomes are either human casualties or property damage [28]. However, in the case of Cyprus, this non-coordinated and fragmented nature of information results in an inconsistent assessment regarding the direct and indirect contribution of roads and roadkill to biodiversity loss. Due to the absence of large wildlife on the island, most roadkill result in minor property damage, if any, that is handled by the insurance companies without any reporting back to the authorities. In most cases animals are just run over by vehicles without an actual “collision” that would justify the report of a WVC. Only a handful of serious life-threatening WVC have been recorded by the police and the impact of small animals on road safety has been downplayed. This despite the fact that small animals on the road surface can be equally dangerous as large ones and may cause an accident if a driver swerves to avoid collision [98,99].

The above issues of the current roadkill systematic monitoring effort conducted by the PWD are currently being addressed in a recent effort, the Cyprus Road Observation System – CyROS (www.cyroadkills.org). CyROS aims to improve the information provided in collaboration with government departments and in conjunction with citizen science data, providing new insights into roadkill distribution, hotspots and their persistence over time [37].

## 5 Conclusions

This is the very first account of roadkill from Cyprus with the use of a systematic long-term dataset. Identifying the main roads where most roadkill occur and the association with the phenomenon species are important parameters for biodiversity conservation and road safety. There is a clear need to improve monitoring protocols and provide training for animal identification to the agencies responsible for data collection on the island. However, and despite the limitations reported herein, the collected information can contribute towards mitigating the phenomenon, improving protected species and areas management, and road safety. Given the pace of road construction on the island and the fact that roadkill are not currently taken into consideration during the road design/ construction phase, the evaluation of the longest systematic scheme of roadkill records on the island, the identification of drawbacks, opportunities, and possibilities for its improvement, is of utmost importance.

## Supporting information

S1 Table Number of roadkill recorded yearly, by the PWD, during the 10-year period (2013–2022). Roadkill incidents are shown by animal group (taxon) and in total. NIA = Not-Identified Animal.
(DOCX)

S2 Table Number of roadkill recorded yearly per district, by the PWD, during the 10-year period (2013–2022). (DOCX)

S3 Table Type and number of birds identified as roadkill by the PWD, during the 10-year period (2013–2022). (DOCX)

S1 Fig An overview of Cyprus road network. Existing highways (red lines), of apr. 271km, are to be expanded by 45% until 2050. The new network (blue lines), currently under design, will cover apr. 122 km.
(DOCX)

S2 Fig Total number roadkill and number of roadkill per km per year on nine of the 27 roads monitored by the PWD, during the 10-year period (2013–2022). These nine roads are the roads where most roadkill incidents were recorded.
(DOCX)
