# Supplementary material for: Roadkill in a Mediterranean island: Evaluating ten-years of official records
Source: PLoS One. 2025 May 20;20(5):e0322644. doi: 10.1371/journal.pone.0322644 (PMC12092012; doi:10.1371/journal.pone.0322644)
Supplement: S1 Table — Roadkill incidents are shown by animal group (taxon) and in total. NIA = Not-Identified Animal. (DOCX) [file pone.0322644.s001.docx]

**Supporting information – Table S1**

**Table S1**: Number of roadkill recorded yearly, by the PWD, during the 10-year period (2013-2022). Roadkill incidents are shown by animal group (taxon) and in total. NIA= Not-Identified Animal.

| **Year** | **Taxon** | | | | | | **NIA** | **Total** |
| --- | --- | --- | --- | --- | --- | --- | --- | --- |
|  | **Fox** | **Bird** | **Hedgehog** | **Snake** | **Rat** | **Hare** |  |  |
| **2013** | 48 | 127 | 55 | 17 | 24 | 2 | 9 | 282 |
| **2014** | 72 | 126 | 72 | 31 | 19 | 5 | 8 | 335 |
| **2015** | 80 | 78 | 97 | 58 | 12 | 2 | 28 | 355 |
| **2016** | 102 | 31 | 1 | 9 |  | 2 | 68 | 213 |
| **2017** | 85 | 20 |  | 14 |  | 1 | 23 | 143 |
| **2018** | 56 | 3 |  | 3 |  |  | 6 | 68 |
| **2019** | 39 |  |  | 1 |  |  | 10 | 50 |
| **2020** | 93 | 29 |  | 7 |  | 5 | 1 | 135 |
| **2021** | 132 | 48 |  |  |  | 4 |  | 184 |
| **2022** | 155 | 60 |  |  |  | 1 | 4 | 220 |
| **Total** | **862** | **522** | **225** | **140** | **55** | **22** | **157** | **1,985** |
