# Supplementary material for: Roadkill in a Mediterranean island: Evaluating ten-years of official records
Source: PLoS One. 2025 May 20;20(5):e0322644. doi: 10.1371/journal.pone.0322644 (PMC12092012; doi:10.1371/journal.pone.0322644)
Supplement: S2 Table — (DOCX) [file pone.0322644.s002.docx]

**Supporting information – Table S2**

**Table S2**: Number of roadkill recorded yearly per district, by the PWD, during the 10-year period (2013-2022).

| **Year** |  |  | **Districts** |  |  | **Total** |
| --- | --- | --- | --- | --- | --- | --- |
|  | **Nicosia** | **Limassol** | **Larnaca** | **Pafos** | **Famagusta** |  |
| 2013 | 282 |  |  |  |  | 282 |
| 2014 | 299 | 11 | 20 |  | 5 | 335 |
| 2015 | 314 | 16 | 25 |  |  | 355 |
| 2016 | 134 | 25 | 52 |  | 2 | 213 |
| 2017 | 63 | 20 | 44 | 15 | 1 | 143 |
| 2018 | 10 | 21 | 22 | 12 | 3 | 68 |
| 2019 |  | 21 | 22 | 6 | 1 | 50 |
| 2020 | 10 | 11 | 100 | 14 |  | 135 |
| 2021 | 78 | 17 | 78 | 11 |  | 184 |
| 2022 | 107 | 17 | 87 | 8 | 1 | 220 |
| **Total** | **1,297** | **159** | **450** | **66** | **13** | **1,985** |
