# Supplementary material for: Roadkill in a Mediterranean island: Evaluating ten-years of official records
Source: PLoS One. 2025 May 20;20(5):e0322644. doi: 10.1371/journal.pone.0322644 (PMC12092012; doi:10.1371/journal.pone.0322644)
Supplement: S3 Table — (DOCX) [file pone.0322644.s003.docx]

**Supporting information – Table S3**

**Table S3**: Type and number of birds identified as roadkills by the PWD, during the 10-year period (2013-2022).

| **Type of bird** | **Number of roadkill recorded** |
| --- | --- |
| Dove / pigeon | 157 |
| Owl | 58 |
| Hooded crow | 51 |
| Falcon | 19 |
| Partridge | 18 |
| Francolin | 6 |
| Sparrow | 6 |
| Eurasian magpie | 4 |
| Parrot | 3 |
| Hoopoe | 1 |
| Duck | 1 |
| Goose | 1 |
| **Total** | **325** |
