# Supplementary material for: Roadkill in a Mediterranean island: Evaluating ten-years of official records
Source: PLoS One. 2025 May 20;20(5):e0322644. doi: 10.1371/journal.pone.0322644 (PMC12092012; doi:10.1371/journal.pone.0322644)
Supplement: S1 Fig — Existing highways (red lines), of apr. 271km, are to be expanded by 45% until 2050. The new network (blue lines), currently under design, will cover apr. 122 km. (DOCX) [file pone.0322644.s004.docx]

**Supporting information – Figure S1**

**
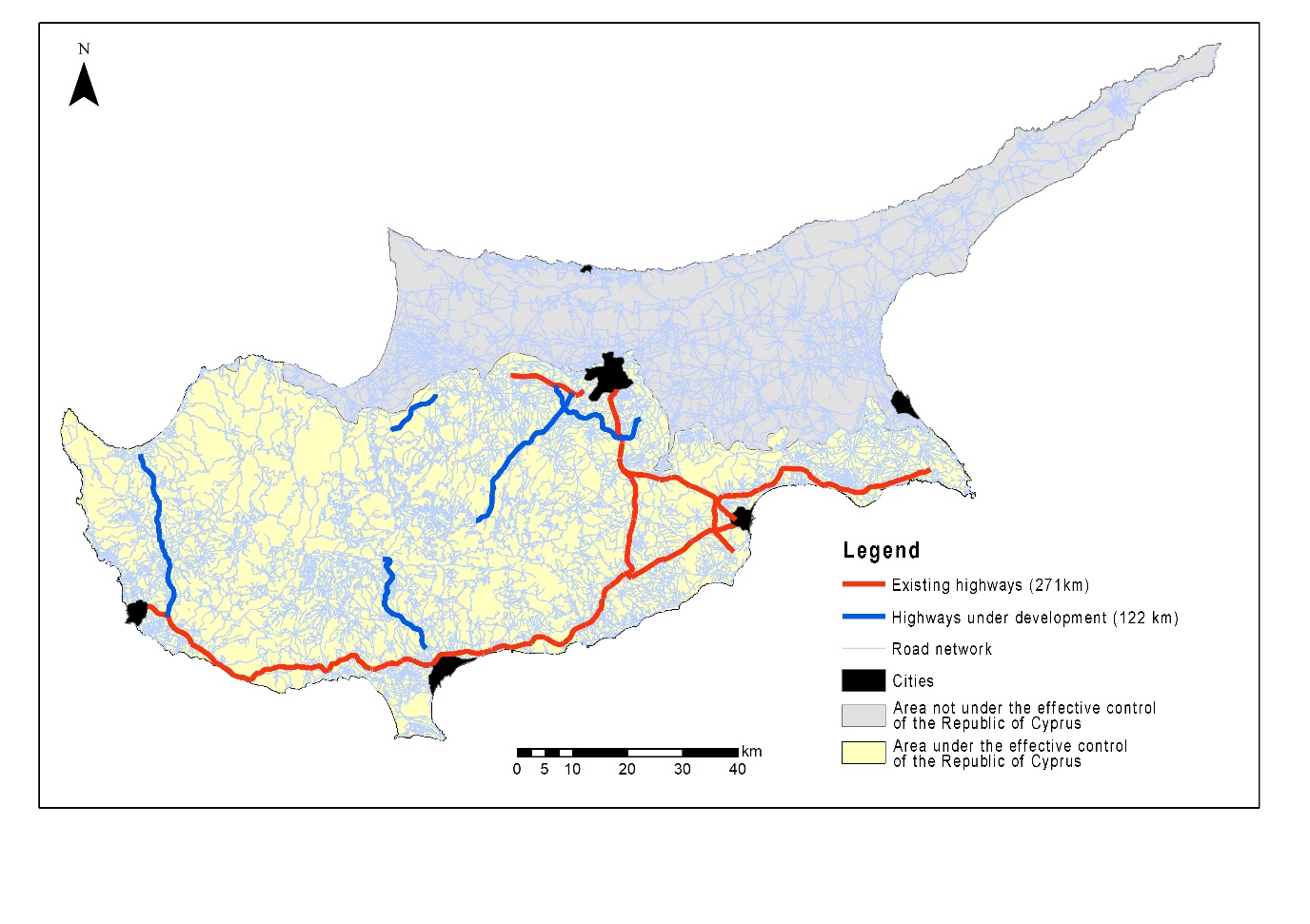
**

**Figure S1.** An overview of Cyprus road network. Existing highways (red lines), of apr. 271km, are to be expanded by 45% until 2050. The new network (blue lines), currently under design, will cover apr. 122 km.
