# Supplementary material for: Roadkill in a Mediterranean island: Evaluating ten-years of official records
Source: PLoS One. 2025 May 20;20(5):e0322644. doi: 10.1371/journal.pone.0322644 (PMC12092012; doi:10.1371/journal.pone.0322644)
Supplement: S2 Fig — These nine roads are the roads where most roadkill incidents were recorded. (DOCX) [file pone.0322644.s005.docx]

**Supporting information – Figure S2**


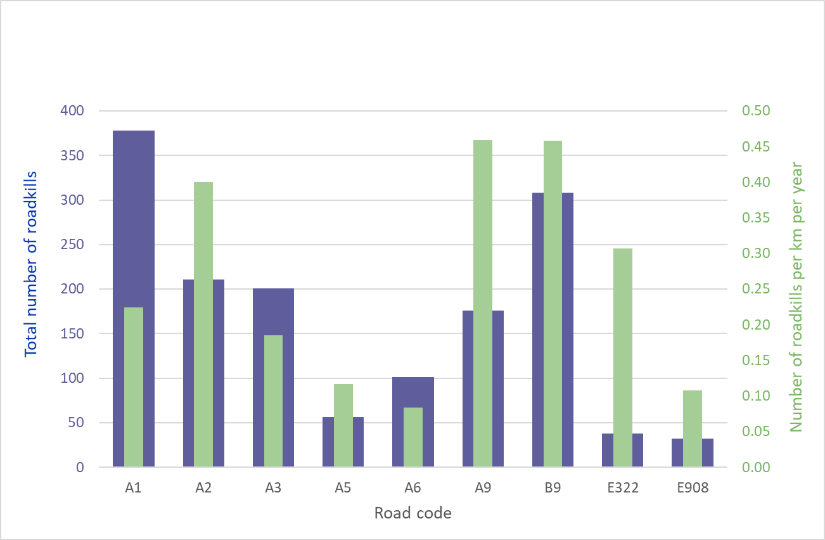


**Figure S2.** Total number roadkills and number of roadkills per km per year on nine of the 27 roads monitored by the PWD, during the 10-year period (2013-2022). These nine roads are the roads where the more roadkills were recorded.
